# The relationships among individual and regional smoking, socioeconomic status, and oral and pharyngeal cancer survival: a mediation analysis

**DOI:** 10.1002/cam4.509

**Published:** 2015-08-07

**Authors:** Yi Guo, Henrietta L Logan, John G Marks, Elizabeth A Shenkman

**Affiliations:** 1 Department of Health Outcomes and Policy, University of FloridaGainesville, Florida; 2 Department of Community Dentistry and Behavioral Science, University of FloridaGainesville, Florida

**Keywords:** Cancer survival, mediation analysis, oral and pharyngeal cancer, smoking, socioeconomic status

## Abstract

Poorer survival from oral and pharyngeal cancer (OPC) has been reported for populations of lower socioeconomic status (SES), adjusting for risk factors such as patient and clinical characteristics. Beyond these risk factors, higher rates of tobacco use may be a mediator for the observed poorer OPC survival for low SES populations. In this study, we aimed to examine the impact of the relationships among SES, individual smoking status, and living in a region with a higher smoking rate on OPC survival. We obtained Florida Cancer Data System data from 1996 to 2010 and merged the data with US Census data and Behavioral Risk Factor Surveillance System data from 1996 to 2010. We built multivariable survival models to quantify the mediational effect of individual smoking on overall and OPC-specific survival, adjusting for regional smoking, demographics, and clinical characteristics. We found that lower SES, individual smoking, and living in a region with a higher smoking rate were all strongly associated with poorer survival. We estimated that the indirect effect of individual smoking accounted for a large part (ranged from 13.3% to 30.2%) of the total effect of SES on overall and OPC-specific survival. In conclusion, individual and regional smoking are both significant and independent predictors of poor cancer survival. Higher rate of individual smoking is partially responsible for poorer cancer survival in low SES populations. Results of this study provide rationale for considering a multi-level approach that simultaneously targets both individual and contextual factors for future smoking cessation interventions.

## Introduction

Oral and pharyngeal cancer (OPC), or head and neck cancer, is a deadly disease with high morbidity that disproportionally affects low socioeconomic status (SES) populations 1. Prior research has documented that OPC patients of lower SES have poorer survival compared to those of higher SES 2–5. One major reason for the poorer survival of low SES patients is that these individuals are more likely to present with advanced-stage cancer 3. However, many studies show that the disparity in cancer survival by SES is still present even after adjusting for tumor stage, as well as other demographic and clinical factors 4. For example, in one study of cancers of the oral cavity, pharynx, and larynx, the authors found that the low SES group had significantly poorer cancer-specific survival than the high SES group, after adjusting for stage of diagnosis, tumor grade, treatment, hospital type, and patient characteristics 4. The findings suggest that, beyond demographics and clinical factors, other significant factors are also contributing to the poorer cancer outcomes among low SES populations.

One potential factor that could explain the poorer OPC outcomes among populations of lower SES may be higher rates of high-risk behaviors such as tobacco use, which appears to be the main cause of OPC and many other types of cancers 1,6. In fact, tobacco use has been associated with increased risk of death for cancer patients diagnosed with OPC 7. In a matched-pair analysis of survival among patients diagnosed with head and neck cancer, the investigators found that patients who had smoked (current or former smokers) had significantly higher risk of overall death, head and neck cancer-specific death, and cancer recurrence than those who had never smoked 8. This association between smoking and poor OPC survival is not surprising. Evidence suggests that OPC tumors from smokers have molecular changes not found in those of patients who have never smoked, which may lead to a worse prognosis for smokers 8. In addition, research shows that smoking can interfere with OPC treatments 9. It has been reported that OPC patients who smoked during radiation therapy had significantly lower rates of tumor response to treatment and shorter survival than those who did not smoke 10. In short, negative outcomes from smoking are strongly associated with all aspects of OPC from inception to survival.

**Figure 1:**
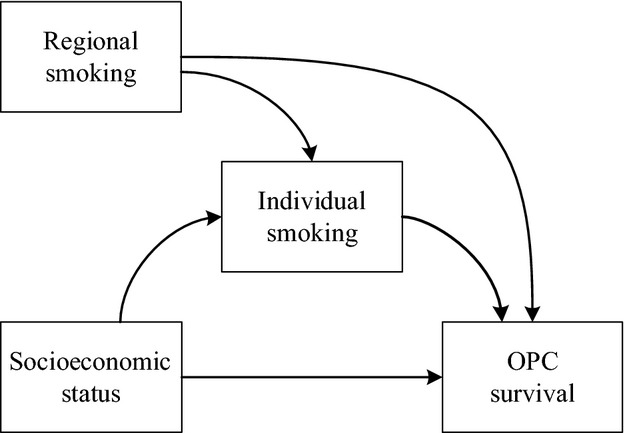
Conceptualized and tested mediation model of how SES and smoking impact OPC survival. SES, socioeconomic status; OPC, oral and pharyngeal cancer. Other control variables included age of diagnosis, gender, race-ethnicity, marital status, insurance, year of diagnosis, anatomic site, stage of diagnosis, and treatment.

In the current study, we hypothesized that low SES and smoking would be associated with poorer OPC survival. We were interested in individual smoking status as well as smoking rates in the region where the individual resided. Our rationale was that smoking behavior is driven by both the biological addiction as well as the social milieu of the individual 11. We reasoned that living in a region with a higher smoking rate might be a surrogate for additional risk since higher instances of regional smoking might lead individuals to maintain or intensify smoking behavior 12 as well as to a higher exposure to the damaging byproducts of smoking 13. Furthermore, we hypothesized that individual smoking mediated the relationship between low SES and poor OPC survival. We built a survival model to examine the impacts of SES and both individual and regional smoking on OPC survival, controlling for demographics and clinical characteristics. Then, based on the survival model, we quantified the mediational effect of individual smoking on OPC survival, while controlling for regional smoking, demographics, and clinical characteristics (Fig.1). In the mediation analysis, we calculated the proportions of total differences in cancer survival across SES groups that could be attributed to individual smoking. Although prior research has examined the association between SES and cancer survival, we are aware of no study that measured the mediational effect of individual smoking in explaining the differences in cancer survival across SES groups.

## Methods

### Data source and case selection

We obtained patients' demographic, tumor, treatment, and survival information from the 1996–2010 data of Florida Cancer Data System (FCDS), a statewide population-based registry supported by the Florida Department of Health and the Centers of Disease Control and Prevention 14. We obtained census tract-level poverty information from the 2000 U.S. census data 15, and also obtained 1996−2010 county-level smoking rates from the Behavioral Risk Factor Surveillance System (BRFSS) of the Centers for Disease Control and Prevention 16.

We included patients who were diagnosed with OPC and were at least 20 years old at the time of diagnosis. Primary anatomic sites were identified using International Classification of Disease (ICD-O-3) codes C00.0 through C14.8. We excluded patients diagnosed before 1996 when type of health insurance was not routinely recorded in FCDS. A total of 25,157 patients, diagnosed between 1996 and 2010, were included in the final sample.

### Variable definitions

We defined survival time (in months) as the time from the date of diagnosis to the date of death or the date of last contact. We grouped anatomic sites into oral squamous cell carcinoma (SCC), pharyngeal SCC, and other head and neck cancers 17. Oral SCC sites were tongue (C02.0–C02.9), floor of mouth (C04.0–C04.9), and palate (C05.0–C05.9). Pharyngeal SCC sites were base of tongue (C01.9), tonsil (C09.0–C09.9), hypopharynx (C12.9–C13.9), oropharynx (C10.0–C10.9). Other head and neck sites were salivary (C07.9–C08.9) and nasopharynx (C11.0–C11.9). Stage of diagnosis was defined based on the Surveillance, Epidemiology, and End Results (SEER) summary stage information and classified as local, regional, distant, or unstaged.

Individual smoking was measured as the cigarette smoking status recorded at time of diagnosis. We grouped patients into smokers (former or current smokers) and nonsmokers. Regional smoking was measured as the average percentage of adult current smokers at the county level between 1996 and 2010. The percentages of adult current smokers were grouped into: ≤15.0%, 15.1–20.0%, 20.1–25.0%, and ≥25.1%.

We defined SES based on the percentage of the population living below the federal poverty level. Use of census tract poverty level as a measure of SES is based on an extensive amount of research by Krieger et al. 18–20, and census tract poverty level has been shown to be a good measure of SES. We linked each patient's FCDS record with Census 2000 data. According to poverty level, we categorized the patients into: high SES (0–9.9% poverty rate), middle SES (10.0–19.9% poverty rate), and low SES (≥20.0% poverty rate).

### Statistical analysis

Differences in distribution of variables of interest by SES were tested with the chi-square test. The association between the predictor variables and OPC survival (overall and OPC-specific survival) was examined through multivariable Cox regression modeling. In the survival model, included predictor variables were chosen to represent influences from patient demographic characteristics (age at diagnosis, gender, race-ethnicity, marital status, health insurance, and SES), clinical and tumor characteristics (year of diagnosis, anatomic site, stage of diagnosis, and treatment), and smoking behavior (individual and regional smoking status). We evaluated the proportional hazard assumption by testing the significance of time-dependent covariates (interactions of predictors and log function of survival time) in the model and by visually examining log(−log) survival curves for parallelism.

Quantification of the mediational effects of individual smoking while controlling for regional smoking and the other confounders was performed using a recently developed and validated method for mediation analysis in the survival context 21,22. We decomposed the total effect of SES on OPC survival into direct effect and indirect effect through individual smoking 21. In the analysis, we controlled for regional smoking, age at diagnosis, gender, race, ethnicity, marital status, health insurance, year of diagnosis, anatomic site, stage of diagnosis, and treatment type. The total effect was the difference in OPC survival between the SES levels that was unexplained by the control variables. The indirect effect was the part of the unexplained effect (total effect) that could be attributed to individual smoking. The direct effect was the part of the unexplained effect (total effect) that was left unexplained by individual smoking. The direct and indirect effects were computed as difference in the number of cancer cases per unit of time 21. We performed the mediation analysis using statistical software R version 3.0.3 23. We performed all the other analyses using SAS software version 9.4 (SAS, Cary, NC).

## Results

### Differences in demographics by SES

We summarized the distribution of predictor variables by SES in Table1. There was a significantly higher percentage of non-Hispanic blacks in the low SES group (32.1%) compared to the middle (7.4%) and high SES (2.4%) groups. Patients in the high SES group (31.2%) were more likely to have private insurance than those in the middle (26.3%) and low SES (23.4%) groups. In contrast, patients in the low SES group (16.3%) were more likely to have Medicaid than those in the middle (8.9%) and high (3.9%) SES groups. Regarding smoking-related variables, we observed a significantly increasing percentage of smokers across the high (75.4%), middle (80.0%), and low (82.1%) SES groups. Furthermore, low SES patients were significantly more likely to live in areas with greater smoking rates. For example, only 6.0% of the low SES patients lived in areas with the lowest (≤15.0%) smoking rate. In contrast, the percentage of high SES patients who lived in areas with the lowest smoking rate was 29.2%.

**Table 1:** Distribution of patient characteristics by SES, 1996–2010

Variable	High SES, % (*n* = 12,983)	Middle SES, % (*n* = 8069)	Low SES, % (*n* = 3871)	*P*
Age at diagnosis				
20−44	6.6	7.4	8.3	<0.001
45−64	45.8	49.9	54.3	
64+	47.6	42.7	37.4	
Gender				
Female	30.9	29.3	27.2	<0.001
Male	69.1	70.7	72.8	
Race-ethnicity				
White, non-Hispanic	91.2	81.0	49.6	<0.001
Black, non-Hispanic	2.4	7.4	32.1	
Hispanic	6.4	11.6	18.3	
Marital status				
Married	61.2	52.2	38.2	<0.001
Single	36.3	43.9	57.9	
Unknown	2.5	3.9	3.9	
Insurance				
Private	31.2	26.3	23.4	<0.001
Uninsured	4.7	7.5	10.0	
Medicaid	3.9	8.9	16.3	
Medicare	15.3	16.7	18.4	
Medicare with supplement	27.8	22.4	16.4	
Other	13.4	14.3	11.8	
Unknown	3.6	4.0	3.8	
Year of diagnosis				
1996−2000	31.2	32.5	36.7	<0.001
2001−2005	28.9	28.6	27.1	
2006−2010	39.9	38.9	36.2	
Anatomic site				
Oral SCC	31.2	31.7	32.0	0.106
Pharyngeal SCC	52.5	53.0	53.4	
Other head and neck sites	16.3	15.3	14.7	
Stage of diagnosis				
Local	29.2	27.0	22.4	<0.001
Regional	48.3	49.0	50.9	
Distant	10.0	11.9	15.2	
Unstaged	12.5	12.1	11.5	
Treatment				
Surgery only	27.0	24.9	19.0	<0.001
Surgery + radiation and/or chemo	27.0	24.7	22.8	
Radiation and/or chemo only	35.5	37.2	41.9	
Unknown	10.5	13.2	16.3	
Individual smoking				
Nonsmokers	24.6	20.0	17.9	<0.001
Smokers (former or current)	75.4	80.0	82.1	
Regional smoking				
≤15.0%	29.2	13.0	6.0	<0.001
15.1–20.0%	24.4	25.8	33.9	
20.1–25.0%	39.4	48.8	52.1	
≥25.1%	7.0	12.4	8.0	

SES, socioeconomic status; SCC, squamous cell carcinoma.

### Overall and OPC-specific survival

We summarized the results from the multivariable Cox models in Table2. Adjusting for the demographic and clinical characteristics, the hazard of overall death was 1.12 times (95% CI = 1.08–1.17) greater for middle SES patients and 1.26 times (95% CI = 1.20–1.33) greater for low SES patients compared to patients in the high SES group. Adjusting for the demographic and clinical characteristics, the hazard of OPC-specific death was 1.18 times (95% CI = 1.11–1.25) greater for middle SES patients and 1.37 times (95% CI = 1.26–1.49) greater for low SES patients compared to patients in the high SES group.

**Table 2:** HR from cox regression models of OPC survival

Variable	Overall survivalHR (95% CI)	OPC-specific survivalHR (95% CI)
Age at diagnosis	1.32 (1.30–1.35)	1.39 (1.35–1.43)
Gender		
Female	1.00	1.00
Male	1.10 (1.06–1.14)	1.03 (0.97–1.09)
Race-ethnicity		
White, non-Hispanic	1.00	1.00
Black, non-Hispanic	1.25 (1.17–1.32)	1.23 (1.12–1.36)
Hispanic	0.95 (0.89–1.02)	0.94 (0.84–1.06)
Marital status		
Married	1.00	1.00
Single	1.25 (1.21–1.30)	1.29 (1.22–1.37)
Unknown	1.10 (1.01–1.21)	1.12 (0.96–1.31)
Insurance		
Private	1.00	1.00
Uninsured	1.62 (1.51–1.75)	1.83 (1.63–2.05)
Medicaid	1.63 (1.52–1.75)	1.86 (1.67–2.07)
Medicare	1.27 (1.20–1.34)	1.41 (1.28–1.54)
Medicare with supplement	1.11 (1.05–1.17)	1.12 (1.02–1.22)
Other	1.11 (1.04–1.17)	1.12 (1.02–1.23)
Unknown	1.28 (1.18–1.39)	1.50 (1.31–1.72)
Year of diagnosis		
1996–2000	1.00	1.00
2001–2005	1.10 (1.06–1.14)	0.77 (0.72–0.83)
2006–2010	1.09 (1.04–1.14)	0.58 (0.54–0.62)
Anatomic site		
Oral SCC	1.00	1.00
Pharyngeal SCC	0.87 (0.83–0.90)	0.72 (0.68–0.77)
Other head and neck sites	0.86 (0.82–0.91)	0.75 (0.68–0.81)
Stage of diagnosis		
Local	1.00	1.00
Regional	1.45 (1.38–1.51)	1.82 (1.68–1.97)
Distant	2.40 (2.26–2.55)	3.22 (2.92–3.55)
Unstaged	1.29 (1.22–1.37)	1.62 (1.47–1.79)
Treatment		
Surgery only	1.00	1.00
Surgery + radiation and/or chemo	1.04 (0.99–1.09)	1.17 (1.07–1.28)
Radiation and/or chemo only	1.40 (1.33–1.47)	1.74 (1.60–1.89)
Unknown	1.98 (1.87–2.10)	2.50 (2.26–2.76)
Socioeconomic status		
High	1.00	1.00
Middle	1.12 (1.08–1.17)	1.18 (1.11–1.25)
Low	1.26 (1.20–1.33)	1.37 (1.26–1.49)
Regional smoking		
≤15.0%	1.00	1.00
15.1–20.0%	1.17 (1.10–1.25)	1.39 (1.24–1.56)
20.1–25.0%	1.21 (1.14–1.29)	1.49 (1.33–1.66)
≥25.1%	1.25 (1.15–1.35)	1.56 (1.36–1.79)
Individual smoking		
Nonsmokers	1.00	1.00
Smokers (former or current)	1.36 (1.30–1.42)	1.48 (1.37–1.59)

All chosen predictor variables were included in the final model. HR, hazard ratios; OPC, oral and pharyngeal cancer; SCC, squamous cell carcinoma.

Regarding the effects of tobacco use, individual smoking, and regional smoking were significant and independent predictors of poor cancer survival. Patients who had ever smoked (former or current smokers) had significantly elevated risk of overall (hazard ratios [HR] = 1.36, 95% CI = 1.30–1.42) and OPC-specific (HR = 1.48, 95% CI = 1.37–1.59) death than patients who had never smoked. Furthermore, patients who lived in areas with higher smoking rates had significantly elevated risk of overall and OPC-specific death than those lived in areas with lower smoking rates. The HR increased as regional smoking rate increased for both overall (HR from 1.17 to 1.25) and OPC-specific (HR from 1.39 to 1.56) death.

### Quantifying the effect of individual smoking on cancer survival

Lastly, we computed how much of the total difference in survival across the SES groups could be attributed to individual smoking, while controlling for regional smoking, and demographic and clinical characteristics (Fig.1). The results were summarized in Table3. For the difference in overall survival between middle and high SES patients, the direct effect (10^−4^) was 23.6 (95% CI = 20.0–27.2) and the indirect effect (10^−4^) through the pathway of individual smoking was 10.2 (95% CI = 6.4–14.0). In other words, the difference in overall survival, controlling for regional smoking and demographic and clinical characteristics, was computed to be 33.8 (23.6 + 10.2) more death cases for middle SES patients per month per 10,000 patients. Of the 33.8 cases, 10.2 cases (30.2%) could be attributed to individual smoking. Similarly, compared to high SES patients, there were 55.8 (44.8 + 11.0) more death cases for low SES patients per month per 10,000 patients. Of the 55.8 cases, 11.0 cases (19.7%) could be attributed to individual smoking.

**Table 3:** Decomposition of total effect in the mediation analysis

Socioeconomic status	Direct effect^1^ (95% CI) × 10^−4^	Indirect effect via smoking^1^ (95% CI) × 10^−4^	Percentage of total effect explained, %^2^
Overall survival			
Middle vs. high	23.6 (20.0, 27.2)	10.2 (6.4, 14.0)	30.2
Low vs. high	44.8 (39.0, 50.6)	11.0 (7.1, 14.9)	19.7
OPC-specific survival			
Middle vs. high	17.7 (14.1, 21.3)	4.8 (1.2, 8.4)	21.3
Low vs. high	37.0 (31.1, 42.9)	5.7 (2.1, 9.3)	13.3

OPC, oral and pharyngeal cancer.

1 Adjusted for age of diagnosis, gender, race-ethnicity, marital status, insurance, year of diagnosis, anatomic site, stage of diagnosis, treatment, and regional smoking.

2 Percentage of total effect (difference in OPC survival) explained by ever smoking = indirect effect/(indirect effect + direct effect).

For OPC-specific survival, there were 22.5 (17.7 + 4.8) more death cases for middle SES patients per month per 10,000 patients, compared to high SES patients. Individual smoking was responsible for 4.8 (or 21.3%) of the 22.5 cases. In addition, compared to high SES patients, there were 42.7 (37.0 + 5.7) more death cases for low SES patients per month per 10,000 patients. Individual smoking was responsible for 5.7 (or 13.3%) of the 42.7 cases.

## Discussion

### Principal findings

In this study, we found that individual smoking and greater regional smoking independently predicted poorer cancer survival, adjusting for demographics and clinical characteristics. Controlling for regional smoking, demographics, and clinical characteristics, we estimated that individual smoking accounted for a large part (ranged from 13.3% to 30.2%) of the total difference in overall and OPC-specific survival across the SES groups. In other words, individual smoking was a major reason for poorer OPC survival for low SES populations, beyond the effects of often-reported risk factors including age, gender, race-ethnicity, marital status, insurance, stage of diagnosis, and treatment.

Our findings indicate that smoking cessation is essential for the health outcomes of smokers. Moreover, results from the mediation analysis suggest that the mediation effect of individual smoking is larger in the middle SES group than that in the low SES group. Promoting smoking cessation in general would benefit everyone, but the middle SES group would benefit more from it.

### Policy changes may be necessary to promote smoking cessation

Our results show that individual and regional smoking are simultaneously associated with poor OPC survival. Although a considerable proportion of smokers have tried to quit smoking, the success rates are low 24. Changes in public policies may help better promote smoking cessation, especially among low SES populations. For example, one challenge for those wishing to quit smoking comes from living and working with others who smoke, which may foster nicotine cravings and make quitting difficult 25. In public housing, a service for ensuring availability of housing for low-income populations, there is no federal requirement to adopt smoke-free policies 26. As a result, smokers living in public housing may have greater difficulty quitting because they live with other smokers who are allowed to smoke on-site. For people with discretionary income, moving to smoke-free housing is an option. However, this option is often unavailable for low-income populations. In order to increase quit rate among smokers, greater adoption of smoke-free housing policies should be considered.

Another example is the smoking cessation coverage through Medicaid programs. The Medicaid population may be the most representative of low SES populations. Although 41% of Medicaid enrollees “tried to quit smoking completely” 27, comprehensive evidence-based cessation treatments are not provided by most state Medicaid programs 28. Furthermore, in states where some treatments are covered, Medicaid enrollees are faced with barriers to accessing these treatments, such as copays and limits on the number of covered quit attempts 28 Many state Medicaid programs have been reducing smoking cessation coverage to reduce their cost. However, prior research indicated that providing comprehensive cessation treatments could reduce smoking rates and Medicaid health care costs in the long run 29,30. In Massachusetts, the state Medicaid program heavily promoted its smoking cessation program to healthcare providers and Medicaid enrollees. Later, the state saw a significant decline in smoking rates among Medicaid enrollees and smoking-related health care costs. A cost-benefit analysis revealed that the smoking cessation program resulted in substantial savings for the Medicaid program 30. Adopting comprehensive coverage of evidence-based treatments for nicotine dependency by all state Medicaid programs is a Healthy People 2020 objective (TU-8) 31. Unfortunately, the current status of financial coverage for Medicaid smoking cessation falls well short of this Healthy People 2020 goal 28.

### Limitations

The findings from this study should be interpreted in the context of its limitations. First, individual smoking status was measured at the time of diagnosis. Although patients who were classified as ever smokers had been exposed to the deteriorating effect of smoking, we do not know individual smoking histories (e.g., number of pack-years) in detail. Second, we acknowledge that the use of county level smoking rates as a surrogate for additional risk from smoking is crude. However, the predictive utility of this measure, above and beyond individual smoking at diagnosis, on cancer survival suggests that contextual factors of an individual's environment may also influence his or her cancer health outcomes 32. Finally, we do not have information on other potential risk factors for OPC survival due to the limitation of the dataset. Information on other potential predictors, such as Human Papilloma Virus status, comorbid conditions, and psychosocial factors, were not recorded in the cancer registry and therefore were not analyzed.

## Conclusions

Both Individual smoking and regional smoking affect cancer health outcomes. Results of this study provide rationale for considering a multilevel approach that simultaneously targets both individual and contextual factors for future smoking cessation interventions. Targeting both individual smoking and regional smoking policy simultaneously may create a synergistic effect that could improve the success of cessation programs.

## References

[b1] Goodwin WJ, Thomas GR, Parker DF, Joseph D, Levis S, Franzmann E (2008). Unequal burden of head and neck cancer in the United States. Head Neck.

[b2] Lee CC, Chien SH, Hung SK, Yang WZ, Su YC (2012). Effect of individual and neighborhood socioeconomic status on oral cancer survival. Oral Oncol.

[b3] Conway DI, Petticrew M, Marlborough H, Berthiller J, Hashibe M, Macpherson LMD (2008). Socioeconomic inequalities and oral cancer risk: a systematic review and meta-analysis of case-control studies. Int. J. Cancer.

[b4] Chu KP, Shema S, Wu S, Gomez SL, Chang ET, Le Q-T (2011). Head and neck cancer-specific survival based on socioeconomic status in Asians and Pacific Islanders. Cancer.

[b5] McDonald JT, Johnson-Obaseki S, Hwang E, Connell C, Corsten M (2014). The relationship between survival and socio-economic status for head and neck cancer in Canada. J. Otolaryngol. Head Neck Surg.

[b6] Ward E, Jemal A, Cokkinides V, Singh GK, Cardinez C, Ghafoor A (2004). Cancer disparities by race/ethnicity and socioeconomic status. CA Cancer J. Clin.

[b7] Yu GP, Ostroff JS, Zhang ZF, Tang J, Schantz SP (1997). Smoking history and cancer patient survival: a hospital cancer registry study. Cancer Detect. Prev.

[b8] Pytynia KB, Grant JR, Etzel CJ, Roberts DB, Wei Q, Sturgis EM (2004). Matched-pair analysis of survival of never smokers and ever smokers with squamous cell carcinoma of the head and neck. J. Clin. Oncol.

[b9] Gritz ER, Fingeret MC, Vidrine DJ, Lazev AB, Mehta NV, Reece GP (2006). Successes and failures of the teachable moment: smoking cessation in cancer patients. Cancer.

[b10] Browman GP, Wong G, Hodson I, Sathya J, Russell R, McAlpine L (1993). Influence of cigarette smoking on the efficacy of radiation therapy in head and neck cancer. N. Engl. J. Med.

[b11] Schnoll RA, James C, Malstrom M, Rothman RL, Wang H, Babb J (2003). Longitudinal predictors of continued tobacco use among patients diagnosed with cancer. Ann. Behav. Med.

[b12] Field M, Rush M, Cole J, Goudie A (2007). The smoking Stroop and delay discounting in smokers: effects of environmental smoking cues. J. Psychopharmacol.

[b13] Kashigar A, Habbous S, Eng L, Irish B, Bissada E, Irish J (2013). Social environment, secondary smoking exposure, and smoking cessation among head and neck cancer patients. Cancer.

[b14] Florida Cancer Data System (2014). http://fcds.med.miami.edu/.

[b15] United States Census Bureau (2009). http://factfinder.census.gov/faces/tableservices/jsf/pages/productview.xhtml?pid=ACS_09_5YR_S1701&prodType=table.

[b16] Centers for Disease Control and Prevention (2006). http://www.cdc.gov/brfss/questionnaires/english.html.

[b17] Guo Y, McGorray SP, Riggs CE, Logan HL (2013). Racial disparity in oral and pharyngeal cancer in Florida 1991–2008: mixed trends in stage of diagnosis. Community Dent. Oral Epidemiol.

[b18] Krieger N, Chen JT, Waterman PD, Rehkopf DH, Subramanian SV (2003). Race/ethnicity, gender, and monitoring socioeconomic gradients in health: a comparison of area-based socioeconomic measures—the Public Health Disparities Geocoding Project. Am. J. Public Health.

[b19] Krieger N, Chen JT, Waterman PD, Soobader MJ, Subramanian SV, Carson R (2002). Geocoding and monitoring of US socioeconomic inequalities in mortality and cancer incidence: does the choice of area-based measure and geographic level matter? The Public Health Disparities Geocoding Project. Am. J. Epidemiol.

[b20] Krieger N, Chen JT, Waterman PD, Rehkopf DH, Subramanian SV (2005). Painting a truer picture of US socioeconomic and racial/ethnic health inequalities: the Public Health Disparities Geocoding Project. Am. J. Public Health.

[b21] Lange T, Vansteelandt S, Bekaert M (2012). A simple unified approach for estimating natural direct and indirect effects. Am. J. Epidemiol.

[b22] Lange T, Hansen JV (2011). Direct and indirect effects in a survival context. Epidemiology.

[b23] R Core Team (2014). R Software [computer program].

[b24] (2011). Quitting smoking among adults–United States, 2001-2010. MMWR Morb. Mortal. Wkly Rep.

[b25] Culbertson CS, Bramen J, Cohen MS, London ED, Olmstead RE, Gan JJ (2011). Effect of bupropion treatment on brain activation induced by cigarette-related cues in smokers. Arch. Gen. Psychiatry.

[b26] U.S. Department of Housing and Urban Development (2012).

[b27] Greene J, Sacks RM, McMenamin SB (2014). The impact of tobacco dependence treatment coverage and copayments in Medicaid. Am. J. Prev. Med.

[b28] Centers for Disease Control and Prevention (2014). State medicaid coverage for tobacco cessation treatments and barriers to coverage United States, 2008–2014.

[b29] Land T, Warner D, Paskowsky M, Cammaerts A, Wetherell L, Kaufmann R (2010). Medicaid coverage for tobacco dependence treatments in Massachusetts and associated decreases in smoking prevalence. PLoS One.

[b30] Richard P, West K, Ku L (2012). The return on investment of a Medicaid tobacco cessation program in Massachusetts. PLoS One.

[b31] U.S. Department of Health and Human Services (2014). http://healthypeople.gov/2020/topicsobjectives2020/pdfs/tobaccouse.pdf.

[b32] Probst JC, Moore CG, Glover SH, Samuels ME (2004). Person and place: the compounding effects of race/ethnicity and rurality on health. Am. J. Public Health.

